# The Prince of Wales's General Hospital, Tottenham

**Published:** 1907-05-11

**Authors:** 


### THE PRINCE OF WALES'S GENERAL HOSPITAL, TOTTENHAM.

### THE ROYAL VISIT.

It is with great pleasure that we record the fact that the Prince of Wales has given his name to the Tottenham Hospital, because its rise and development are most interesting facts in the history of modern London. In 1898 the Charity Commissioners altered its constitution which had been established in 1867 as an institution for training nurses on the Kaiserswerth system, with the object of visiting the sick poor in their homes. Under the old constitution it was a home for deaconesses, and the members of the sisterhood rendered aid in nursing patients during epidemics, whilst some of their number acted as nurses in the Franco-German war and found their way to mission stations throughout the world. When the constitution was altered it placed the management in the hands of a committee elected by the Governors, and rendered it possible to make it the nucleus of a new general hospital for the increasing population of Tottenham and the district.

In January 1899 at a public meeting held in Tottenham under the presidency of Sir Henry Burdett, Mr. James Howard, M.P., and the late Dr. T. Gilbart Smith moved a resolution pledging the inhabitants of the district to use every effort to raise the necessary funds to place the hospital on a sound financial basis. There was a large and influential attendance at this meeting, and the Chairman took the opportunity to bring before the inhabitants of the district what had been done by the inhabitants of Highbury, who had raised nearly £100,000, built the Great Northern Central Hospital, provided an income of nearly £10,000 a year, and accumulated in a very short time an invested fund of £30,000. This meeting succeeded in attracting the interest and support of the whole district comprising the inhabitants of Edmonton, Waltham Cross, Waltham Abbey, Southgate, Cheshunt, Wood Green, Ponders End, Clapton, Stoke Newington, Stamford Hill and Enfield. Three years later the Ladies' Association organised a bazaar which was opened by Sir Henry Burdett, and from that time forward the Tottenham Hospital, under the able leadership of Sir Francis Cory Wright, who has shown great generosity and devotion in bringing the work to a successful conclusion, has steadily progressed. At the meeting in 1899 the financial position was lamentable. The institution, with an income of less than £1,400 a year, was expending £5,000, and there were only twenty-four beds available for in-patients. Three years later, when the bazaar was held, the expenditure had been reduced to £4,146, whilst the income had been increased to £4,430, and upwards of seventy beds were available for patients. In 1906 the income had increased to £6,000 in round figures, whilst the expenditure amounted to nearly £7,000. The hospital now contains 123 beds, and the extensions opened by the Prince of Wales include two large wards and a new operation-theatre fully equipped with every modern requirement. To enable the Committee to maintain the whole of these beds an income of not less than £8,500 per annum is essential. That this income will be forthcoming we have little doubt, in view of the success which has attended the excellent business methods pursued by Sir Francis Cory Wright and his Committee during recent years. The hospital ministers to the needs of a population of between 200,000 and 300,000 people.

Another interesting feature connected with the Tottenham development is the fact that, although something like £20,000 has been recently expended upon buildings, equipment and furniture, the whole of this money will be provided, when the £4,000 subscribed on Tuesday is added to the Building Fund. That fund has received no less than £7,000 from King Edward's Hospital Fund and £5,000 from the Drapers' Company, facts which must commend the support of this hospital to those generous givers who contribute to hospitals on business principles. The Drapers' Company has long been noted for the wisdom and generosity which have characterised its donations to public institutions, and we have little doubt that the court will still further aid the Committee by helping them to wipe out the £4,500 of accumulated debt, so as to enable the institution to commence its new career under the most happy auspices. Another source from which a liberal contribution ought to come is the London County Council, for it has been the means of adding some 40,000 souls to the population of the district, by moving people out from congested London areas. The County Council has the power to contribute to hospitals, and it is clearly the duty of this Council, when it causes a movement of the population to the extent indicated, to take care that adequate hospital provision is made for these poor people in the new district thus opened up.

Previous to the arrival of their Royal Highnesses at the Tottenham Hospital on Tuesday the rain came down in torrents, but, so soon as they entered the district, the sun shone forth brightly, and the people flocked out in thousands to welcome the illustrious visitors, and to show by their presence and cheers the cordiality of their gratitude for the splendid help which the Prince of Wales was rendering to the inhabitants of the district in their noble effort to provide adequate hospital accommodation for the comfort and protection of the poorer inhabitants. Their Royal Highnesses never, probably, spent an afternoon to better advantage than in helping the people of this large district to help themselves.

